# Mechanism of calcitriol regulating parathyroid cells in secondary hyperparathyroidism

**DOI:** 10.3389/fphar.2022.1020858

**Published:** 2022-10-04

**Authors:** Zeli Xiang, Ming Wang, Changxiu Miao, Die Jin, Hongyue Wang

**Affiliations:** Department of Nephrology, The First Hospital of Jilin University, Changchun, China

**Keywords:** calcitriol, parathyroid cells, regulatory mechanisms, secondary hyperparathyroidism, chronic kidney disease

## Abstract

A common consequence of chronic renal disease is secondary hyperparathyroidism (SHPT) and is closely related to the mortality and morbidity of uremia patients. Secondary hyperparathyroidism (SHPT) is caused by excessive PTH production and release, as well as parathyroid enlargement. At present, the mechanism of cell proliferation in secondary hyperparathyroidism (SHPT) is not completely clear. Decreased expression of the vitamin D receptor (VDR) and calcium-sensing receptor (CaSR), and 1,25(OH)2D3 insufficiency all lead to a decrease in cell proliferation suppression, and activation of multiple pathways is also involved in cell proliferation in renal hyperparathyroidism. The interaction between the parathormone (PTH) and parathyroid hyperplasia and 1,25(OH)2D3 has received considerable attention. 1,25(OH)2D3 is commonly applied in the therapy of renal hyperparathyroidism. It regulates the production of parathormone (PTH) and parathyroid cell proliferation through transcription and post-transcription mechanisms. This article reviews the role of 1,25(OH)2D3 in parathyroid cells in secondary hyperparathyroidism and its current understanding and potential molecular mechanism.

## Introduction

Secondary hyperparathyroidism (SHPT), caused by disturbed phosphate and calcium homeostasis, is a common consequence of increased glomerular filtration rate (GFR) loss in chronic kidney disease (CKD). Early SHPT induces polyclonal and widespread growth of the parathyroid gland (PTG), followed by nodular and monoclonal proliferation with ongoing uremia-related stimulation (Tominaga et al., 1996). If poorly managed, SHPT progresses and finally results in soft tissue calcification, vascular calcification, and bone disease such as demineralization, loss of structural strength, fractures, and bone pain, all of which has a serious impact on morbidity and mortality.

SHPT is distinguished by enhanced parathyroid hormone (PTH) production, gene transription as well as parathyroid gland enlargement. PTH synthesis and parathyroid gland proliferation are caused by a variety of factors in CKD patients, including hypocalcaemia, hyperphosphatemia, vitamin D deficiency, reduced density of vitamin D receptor (VDR) and calcium-sensing receptor (CaSR) and disturbance of fibroblast growth factor 23 (FGF23)-Klotho axis (Okazaki et al., 1988; Brown et al., 1993; Urakawa et al., 2006). Post-transcriptional processes are also involved in the increase of PTH levels in SHPT (Kilav-Levin et al., 2020). The processes behind parathyroid cell growth in SHPT remain unknown.

**FIGURE 1:**
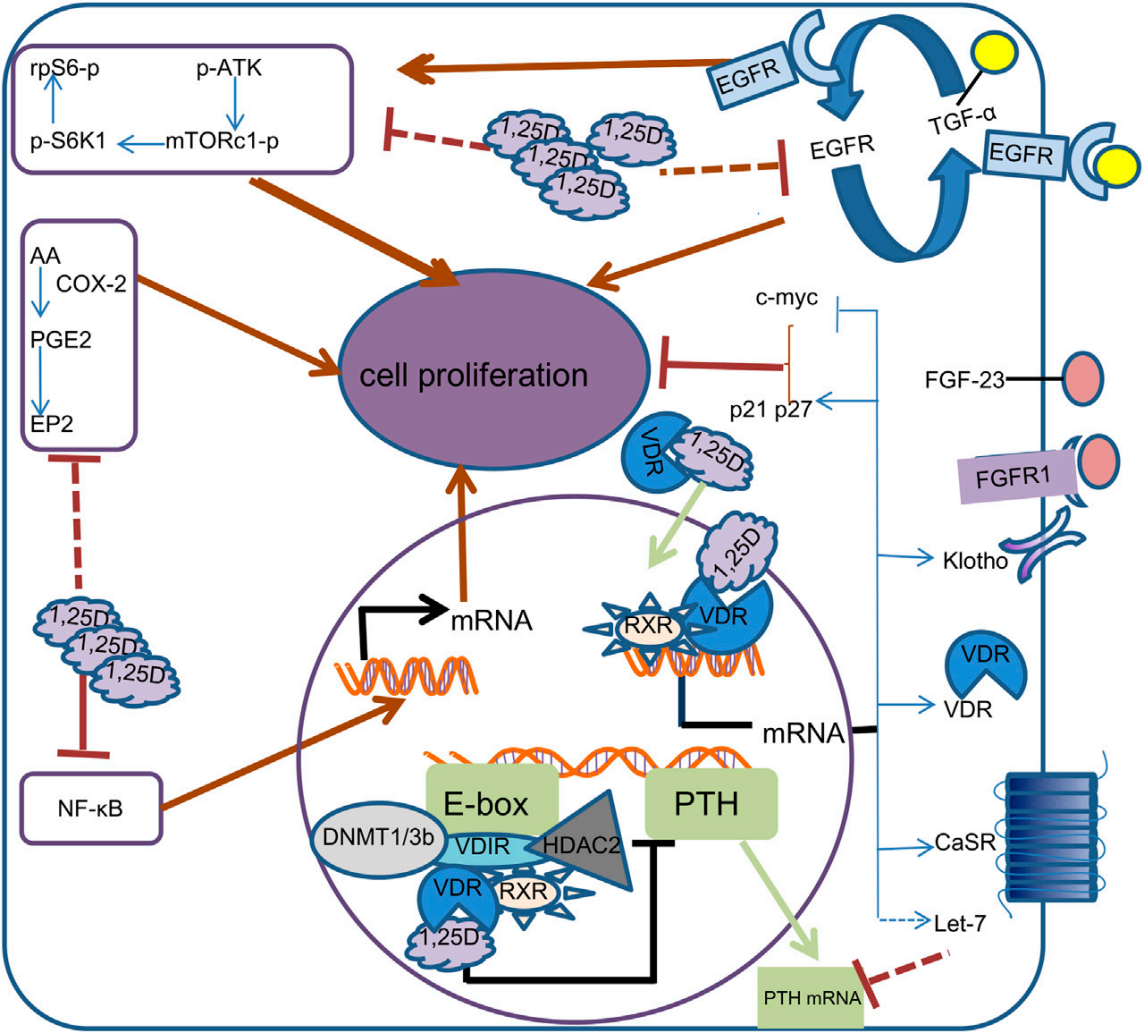
Mechanism of calcitriol in parathyroid cells. In the cell, 1,25D binds to VDR, which is translocated to the nucleus.1, 25D-bound VDR can form different complexes in different promoter regions. By binding to RXR and VDIR, it recruits histone deacetylases HDAC2 and DNA methyltransferases DNMT1 and DNMT3b to inhibit transcription of the PTH gene, thereby reducing the synthesis and secretion of PTH. 1, 25D-bound VDR induces transcription of calcium-sensing receptor (CaSR), Klotho, VDR, Let-7, c-myc, CDKN1a and CDKN1b genes by directly binding RXR to vitamin D response elements on DNA. Increased CaSR expression leads to higher sensitivity of parathyroid cells to calcium, while increased Klotho transcription results in increased sensitivity to FGF23. c-myc, p21 and p27 supress cell proliferation by inhibiting cell cycle progression. The increased transcription of Let-7 family decreased the stability of PTH mRNA. 1,25D also represses the proliferation of Parathyroid cells by reducing epidermal growth factor receptor (EGFR), Nuclear factor-KB (NF-kB), mTOR complex 1(mTORc1) and prostaglandin E2(PGE2) signaling pathways.

Calcitriol (also called 1,25(OH)2D3), frequent active form of vitamin D, which exerts potent anti-proliferation activity through multiple pathways (Kim et al., 2007; Naveh-Many et al., 1990; Canaff & Hendy, 2002; Dusso et al., 2004; Kan*et al.*, 2018), and simultaneously inhibits PTH synthesis and secretion. This article reviews the role of 1,25(OH)2D3 in parathyroid cells in secondary hyperparathyroidism and its current understanding and potential molecular mechanism, with the hope of providing new strategies and ideas for the treatment of secondary hyperparathyroidism (Figure 1).

### 1,25(OH)2D3 inhibits the PTH gene transcription

1,25(OH)2D3 controls PTH concentrations by inhibiting PTH gene expression (Russell et al., 1999). A conserved E-box-type region was found in the PTH promoter by Kim and others, which was required for VDR and RXR to lead to transcriptional suppression of the PTH gene. This E-box could not be bound directly by VDR or RXR. VDR interacting repressor (VDIR) mediates the transcription of PTH gene by VDR–RXR complex through binding with E-box, because this interaction recruits transcription inhibitors like histone deacetylase 2 (HDAC2) and p300 (Kim et al., 2007).

### 1,25(OH)2D3 enhances VDR transcription in parathyroid cells

The reduction of VDR in renal hyperparathyroidism was first reported by Korkor, who found that VDR levels in hypertrophic parathyroid glands in patients with CKD were substantially lower than those in transplant patients, especially when compared with adenomas in patients with primary hyperparathyroidism (PHPT) (Korkor, 1987). Similarly, other researchers have found reductions in VDR proteins in chronic uremic patients (Fukuda et al., 1993). Interestingly, VDR distribution was incredibly unbalanced, with nodules having much lower VDR levels than widespread hyperplasia tissues (Fukuda et al., 1993). It has been proven by Naveh-Many T and others that 1,25(OH)2D3 has the ability to enhance VDR transcriptional activity in parathyroid cells (Naveh-Many et al., 1990), resulting in a rise in VDR protein. In addition, the combination of 1,25(OH)2D3 with VDR directly lengthens the half-life of VDR protein by preventing VDR proteasome degradation (Wiese et al., 1992). This ligand-dependent receptor elevation causes an increased inhibitory impact of 1,25(OH)2D3 on PTH gene, which explains why 1,25(OH)2D3 has such a big influence on PTH gene. However, glands with nodular hyperplasia, which exhibit a lower VDR level than diffuse hyperplasia, are frequently resistant to treatment with 1,25(OH)2D3 pulses (Fukuda et al., 1993).

### 1,25(OH)2D3 increases CaSR level in parathyroid cells

CaSR is a member of the C GPCR family, which plays a crucial role in Ca^2+^ regulation (Brown, 2013). CaSR dysfunction contributes to the advancement of SHPT in CKD patients. Thus, hypocalcaemia, which results from hyperphosphataemia and diminished 1,25(OH)2D3 synthesis in CKD, disables the parathyroid CaSR, elevating PTH secretion. In addition, CaSR has been observed as a phosphate receptor in the parathyroid gland, and hyperphosphatemia, as a non-competitive inhibitor, inhibits its activity, thereby enhancing PTH production (Centeno et al., 2019). Thus, the CaSR is essential for inhibiting parathyroid hyperplasia. At the very least, 1,25(OH)2D3 is an essential factor in regulating parathyroid CaSR transcription. Study has shown that 1,25(OH)2D3 can increase the expression of CaSR in the parathyroid, thyroid, and kidney by binding to RXR/VDR on VDREs in the CaSR gene promoter, P1 and P2, thus upregulating transcription (Canaff & Hendy, 2002). Functional vitamin D response elements (VDREs) can be found in human CaSR gene promoters P1 and P2 (Canaff & Hendy, 2002). CaSR up-regulation by 1,25(OH)2D3 in the parathyroid gland increases the gland’s sensitivity to extracellular Ca^2+^, thereby reducing PTH secretion.

### 1,25(OH)2D3 affects Klotho transcription in parathyroid cells

Klotho is a protein that crosses the cell membrane and is highly expressed in distal convoluted tubules and parathyroid glands (Galitzer et al., 2008). When used as a transmembrane protein, Klotho is a co-receptor for fibroblast growth factor 23 (FGF23), which together with the fibroblast growth factor receptors (FGFRs) plays a role in inhibiting PTH production and secretion (Galitzer et al., 2008). A more severe decrease of Klotho in nodular hyperplasia has been reported (Komaba et al., 2010; Latus et al., 2013). However, whether there is a difference in Klotho expression between nodular and diffuse hyperplastic glands and the regulatory effect of 1,25(OH)2D3 on Klotho has not been elucidated. According to a recent study, loss of CaSR in PTG causes serum PTH rise and PTG hyperplasia, which is exacerbated by additional deletion of Klotho in PTG. This shows that Klotho plays a vital function in suppressing the generation of PTH and the development of PTG in the lack of CaSR. At the same time, *in vivo* PTG cultivation demonstrated that changes in calcium levels had little effect on Klotho expression, suggesting that the decrease in Klotho expression *in vivo* was primarily attributable to CaSR deactivation (Fan et al., 2018). Therefore, up-regulation or activation of Klotho can be used as a therapeutic approach to regulate circulating PTH levels and parathyroid hyperplasia. There are conflicting reports of active vitamin D regulating Klotho expression in uremic tissues (Hofman-Bang et al., 2010; Krajisnik et al., 2010). Therefore, it is important to assess the influence of vitamin D on Klotho in parathyroid glands.

### Antiproliferative effects of 1,25(OH)2D3 on parathyroid cells

The CDKN1a gene and CDKN1b gene encode cyclin dependent kinase inhibitors (CKIs), p21 and p27, which act as regulators to regulate the transition from G1 to S phase of the cell cycle. Low expression of p21 was seen in parathyroid glands of rats with experimental uremia and SHPT (Dusso et al., 2001). According to a study, the low expression of VDR-dependent p21 and p27 may be a crucial contributor in nodular parathyroid gland formation in advanced SHPT (Tokumoto et al., 2002). Cozzolino M et al. found increased p21 expression in hyperplastic parathyroid glands in an early uremia model with vitaminD3 exposure (Cozzolino et al., 2001). The products of CDKN1a gene -induced cell cycle arrest and differentiation are probably the most numerous of these anti-proliferative effects exerted by diverse 1,25(OH)2D3 target genes. However, the molecular basis of 1,25(OH)2D3 actions on p21 transcription has not been understood clearly in the hyperplastic parathyroid glands with CKD. Interestingly, Anna et al. demonstrated functional VDREs in the p21 promotor in MCF-7 human breast cancer cells (Saramäki et al., 2006). However, c-myc (a proto-oncogene) transcription is also significant for promoting cell proliferation. 1,25(OH)2D3 inhibits parathyroid c-myc expression and delays subsequent proliferation in uremic rats (Fukagawa et al., 1991). It is an important gene in cell cycle regulation and is activated by the WNT/ßcatenin pathway, which is overexpressed in colorectal cancer (CRC) and many other types of cancer. The c-myc gene is repressed by 1,25(OH)2D3, both by combining with its promoter through VDR (Toropainen et al., 2010) and by restraining the WNT/ßcatenin signal pathway (Pálmer et al., 2001). Although these cells do not originate from parathyroid tissue, the promotion of p21 production and the inhibition of c-myc transcription by 1,25(OH)2D3 are consistent with those observed in hyperplastic parathyroids. 1,25(OH)2D3 deficiency or a disruption in its action at the parathyroid cell level, both of which are common in uraemic individuals, can reduce c-myc expression inhibition and p21 expression, accelerating cell cycle advancement. Increasing VDR with 1,25(OH)2D3 can correct VDR density, elevate p21 and p27, and inhibit c-myc expression in the parathyroid.

In uremic parathyroid glands, increased co-expression of TGF-α and EGFR leads to aggressive proliferative activity and gland size, exacerbated by high-phosphorus or 1,25(OH)2D3 deficiency (Dusso et al., 2001). Indeed, therapy with 1,25(OH)2D3 can lower TGF-α and AP2 transcription and suppress EGFR activity and parathyroid enlargement in CKD rats (Dusso et al., 2004). In the early stages of CKD, prolonged 1,25(OH)2D3 treatment inhibits growth by blocking EGFR growth signals on the cell membrane and its role in the nucleus as the CCND1 gene (an oncogene, encoding cyclinD1) transactivator, an important factor in human parathyroid hyperplasia (Cordero et al., 2002; Dusso et al., 2004). However, activated EGFR, as a transcription factor, not only activates the CCND1 gene, but reduces VDR mRNA level by 80% in nodular hyperplasia, the most severe form of SHPT, which has the highest resistance to 1,25(OH)2D3 treatment. Signals from TGF-α/EGFR trigger the production of a powerful oncogene, the dominant-negative isoform of the transcription factor C/EBPß (dnC/EBPß, known as LIP). Increasing levels of parathyroid LIP inhibit transcription of the VDR gene by binding to C/EBPß sites in the promoter of the gene (Arcidiacono et al., 2008b). In EGFR-overexpressing A431 cells, 1,25(OH)2D3 causes a shift in the location of TGF-α and EGFR away from the plasma membrane and into the early endocytic compartment. This shift occurs in a VDR-dependent manner, as shown by a considerable elevation of EGFR levels in the cytoplasm of treated cells and a reduction in surface-specific 125I-EGF, all of which down-regulate EGFR-growth signaling and inhibit of cell proliferation. In addition, 1,25(OH)2D3 suppresses the activation and nuclear translocation of ERK1/2 that is triggered by TGF-α/EGFR, and this has a direct correlation with the magnitude of the autocrine TGF-α/EGFR growth ring. The activation and nuclear translocation of ERK1/2 caused by TGF-α/EGFR, which directly correlates with the magnitude of the autocrine TGF-α/EGFR growth ring (Cordero et al., 2002). Activating Protein2a (AP2), which is upregulated by EGFR stimulation and boosts TGF-α expression levels by engaging TGF-α gene promoter, is involved in the creation of this autocrine loop (Arcidiacono et al., 2008a). In addition, 1,25(OH)2D3 disrupts the binding of EGFR to DNA and transactivation of the CCND1 gene promoter, causing cell cycle arrest because activated EGFR can be transferred to the nucleus of EGFR-overexpressed cells and bind to specific DNA sequences to activate transcription of the CCND1 gene (Cordero et al., 2002). The A431 cell did not originate from parathyroid gland, but it displayed a similar EGFR/TGF-α feed-forward circuit to that reported in secondary hyperparathyroidism.

Nuclear factor kappa-B(NF-κB) is able to govern both the cells proliferation and the growth of tumours by directly controlling the expression of a large number of genes associated with cell proliferation, including c-myc, cyclin D1, and p53 (Viatour et al., 2005; Ledoux & Perkins, 2014). Parathyroid hyperplasia may be caused by similar regulation by NF-κB. Previously, activation of NF-κB leads to an increase in the number of proliferating parathyroid cells in rats with uremia (Kan et al., 2018). p65 and p50 come together to form a heterodimer that is put into an inactive state by IκBα in the cytoplasm during the classical stage of the NF-κB signalling cascade. The measurement of p65 phosphorylation is the standard method for determining its level of activity (Perkins, 2007). Mao J, et al. first proof that NF-κB p65 directly promotes PTH expression *via* combined with the p65-responsive region, which is between positions -908 and -897 upstream of the PTH promoter (Mao et al., 2021). The research prompts that activation of NF-κB pathway not only promotes the parathyroid proliferation, but also directly raises the transcription of PTH gene. 1,25(OH)2D3 is known to inhibit NF-κB activation by forming VDR-p65 complex, increases the level of IκBα (Cohen-Lahav et al., 2006), or strengthens the interaction between VDR and IKKβ (Chen et al., 2013), thereby restraining inflammation and tumor growth (Tan et al., 2008). Similar regulation through 1,25(OH)2D3 may underlie the inhibition of parathyroid hyperplasia. However, *in vitro* treatment of PTGs with 1,25(OH)2D3, only a considerable suppression of phos-NF-κB and an elevation of IκBα concentration were seen (Mao et al., 2021), suggesting that 1,25(OH)2D3 restrains the activation of NF-κB pathway by arresting p65 nuclear translocation, blocking NF-κB DNA binding, including PTH gene, increasing IκBα levels, or stabilizing IκBα protein in advanced SHPT of CKD. It is possible that the decreased number of VDR receptors in hyperplastic glands is partially to blame for the decreased inhibitory effect of 1,25(OH)2D3 on the NF-KB pathway.

Multiple lines of evidence imply that cyclooxygenase 2(COX2), its metabolite prostaglandin E2 (PGE2) as well as its receptor EP2 enhance cell growth in SHPT patients with CKD (Zhang et al., 2011; Zhang et al., 2019). This is a potential therapeutic target for secondary hyperparathyroidism. In normal and malignant human prostate cells, 1,25(OH)2D3 significantly inhibited cyclooxygenase-2(COX-2) transcription and translation. Additionally, 1,25(OH)2D3 upregulates the expression of 15-hydroxyprostaglandin dehydrogenase (15-PGDH), which in turn lowers the amount of PGE2 that is produced. This dual effect was coupled with decreased PGE2 secretion in the conditioned medium of 1,25(OH)2D3-exposed prostate cancer cells. 1,25(OH)2D3 decreased the expression of PG receptor EP2 mRNA, suggesting an additional possible mechanism for limiting the bioactivity of PG (Moreno et al., 2005). The same phenomenon was observed in breast cancer cells, where 1,25(OH)2D3 effectively inhibited COX-2 transcription and increased 15-PGDH synthesis (Krishnan et al., 2009). Therefore, it is hypothesized that 1,25(OH)2D3 also inhibits this pathway in a similar manner and exerts an antiproliferative effect in renal parathyrogenic hyperplasia, however, additional *in vitro* and *in vivo* research are required to confirm this hypothesis.

In addition, mTOR complex 1 (mTORc1) is also involved in the proliferation of parathyroid through phosphorylation of rpS6. Inhibition of mTORc1 by rapamycin reduced or inhibited proliferation of parathyroid cells in rats with SHPT and *in vitro* culture of parathyroid organs in uremia rats (Volovelsky et al., 2016). However, little is known about the effect of 1,25(OH)2D3 on this pathway in secondary hyperparathyroidism. As recently shown by Halicka HD and others, the decrease in expression of phosphorylation of mTOR, rpS6 and 4EBP1 was seen in individual TK6 and A549 cell lines treated with vitaminD3 (Halicka et al., 2012). Interestingly, this action was also found *in vitro* and *in vivo* in mammalian cells (Bettoun et al., 2002). Paradoxically, another group reported that 1,25(OH)2D3 dramatically increased the protein phosphorylation levels of Akt, p70S6K, and rpS6 in C2C12 myotubes cultured with leucine + insulin (Salles et al., 2013). mTORc1 detects nutritional and growth elements essential for cell development and proliferation, as is well known (Shimobayashi & Hall, 2014). Thus, mTORc pathway is activated in mice C2C12 myotubes cultured with leucine and insulin, and 1,25 (OH)2D3 further activates this pathway partly by increasing the expression of VDR and IR, increasing phosphorylation of rpS6 and promoting protein synthesis (Salles et al., 2013). However, in Caco-2 cells, 1,25(OH)2D3 enhanced VDR-related Ser/Thr phosphatase activity. This induction resulted in the fast and specific dephosphorylation of p70S6 kinase residues Thr9-389 (p70S6K), leading to inactivation of this kinase (Bettoun et al., 2002). These findings imply that 1,25(OH)2D3 may affect the mTORc pathway in secondary hyperparathyroidism in a varied manner. The impact of 1,25(OH)2D3 on the regulation of the mTORc1 pathway in parathyroid cells must be evaluated in light of the growing spectrum of EGFR/mTORc1/S6K1/rpS6 functions in parathyroid cells. In secondary hyperparathyroidism (SHPT), EGFR activation of the protein kinase B (PKB, commonly named as AKT)-mTORc1 pathway triggers phosphorylation of downstream substrates S6 kinase1 (S6K1) and ribosomal protein S6 (rpS6), leading to parathyroid cell expansion. In hypertrophic parathyroid cells, EGFR overexpression can significantly activate the mTORc1 pathway. 1,25(OH)2D3 may inhibit the EGFR/mTORc1/S6K1/rpS6 pathway through negatively regulating EGFR expression and dephosphorylating S6K1 by VDR to exert anti-proliferation of parathyroid cells, which needs to be verified by *in vivo* and *in vitro* experiments.

Recently, research has shown that microRNAs (miRNAs) participate in regulating PTH mRNA levels by mediating post-transcriptional mechanisms. In CKD, inhibition of a large number of parathyroid let-7 miRNA family and up-regulation of miR-148 members lead to the development of SHP in uremic rats and in parathyroid cultures (Shilo et al., 2017). In developing NKT thymocytes, Pobezinsky et al. found 121 distinct let-7-associated conservative non-coding sequences containing several VDR and RAR binding sequences, and the transcription of let-7 miRNAs is increased at differentiation stage 2 stimulated by active vitamin D (Pobezinsky et al., 2015). Therefore, 1,25(OH)2D3 can enhance let-7 miRNA transcription in a manner of VDR. However, whether 1,25(OH)2D3 has a similar regulatory effect on parathyroid cells in renal parathyroid hyperplasia needs further experimental verification.

Scholars have found that circadian clock genes and downstream cell cycle regulators, Wee1, Cyclin D1, p27, and c-myc, affected by the circadian clock genes, are dysregulated in uremic rats and may be involved in abnormal proliferation of parathyroid cells in secondary hyperparathyroidism (Egstrand et al., 2020). Conditional deletion of the core circadian gene Bmal1 in PTG promotes parathyroid hyperplasia and impairs parathyroid compensatory secretion in uremia rat (Egstrand et al., 2022). Whether deficiency of 1,25(OH)2D3 impacts the transcription of the circadian clock genes is poorly documented in secondary parathyroids.

It is well known that CaSR and VDR expression is considerably decreased in severe secondary hyperparathyroidism, but interestingly, no difference in mRNA levels was detected between diffuse hyperplasia and nodular hyperplasia (Santamaría et al., 2005), suggesting that the decrease in protein levels is mainly due to post-transcriptional regulation. Due to the expression of genes disorder, the stability of damaged DNA and RNA, protein synthesis and processing impeded (Santamaría et al., 2005), kidney secondary hyperparathyroidism in late to become independent, so overproduction of PTH is no longer to respond to a physiological stimulus or positive drug treatment, at this moment need invasive surgery such as parathyroidectomy.

## The local activation of vitamin D within parathyroid glands

Compared with the normal gland, the mRNA expression and protein level for 1α-hydroxylase (CYP27B1) in secondary hyperplastic parathyroid cells is higher (Segersten et al., 2002). An increase in 1α-hydroxy enzyme concentration (about 10-fold) and a decrease in 24-hydroxy enzyme concentration (about 1/10-fold) were found in 78% of secondary proliferative PTG cells, highlighting the requirement for more than 25D in SHPT (Correa et al., 2002). In parathyroid tissues, 25D and 1,25(OH)2D3 bound to DBP can enter cellular tissues by endocytosis of megalin/cuBulin, but not limited to diffusion by free hormones (Bikle et al., 2017). The 1α-hydroxylase metabolizes 25D to the active 1,25(OH)2D3 locally. In previous proteomic studies, intracellular DBP was found to be significantly downregulated in parathyroid cells, especially in parathyroid nodules dominated by oxytropic cells, which is thought to be closely related to 1,25(OH)2D3 resistance in SHPT patients (Lewin et al., 2003).

## Conclusion and future recommendations

In summary, 1,25(OH)2D3 and its analogues effectively inhibit PTH synthesis and cell proliferation through transcriptional and post-transcriptional mechanisms. The biological reaction is mediated by the binding of 1,25(OH)2D3 to intracellular receptors belonging to the steroid receptor superfamily. On the one hand, as ligand-dependent transcription factors, these receptors bind to specific DNA sequences and exert their biological effects. Two vitamin D response elements have been found, which are triggered either alone by VDR or by heterodimers of the vitamin D receptor and the retinoid-X receptor-a (RXR-a) (Carlberg et al., 1993). In addition, a percentage of VDR molecules are positioned in the cytoplasm of many cell types and settings, where they are responsible for mediating ligand-dependent rapid regulatory effects on signalling pathways that influence phosphatases, kinases, enzymes, and ion channels (Hii and Ferrante, 2016). These impacts do not need any modifications to the transcription of genes, hence they are referred to as non-genomic effects. The PRIMO and OPERA Trials have also demonstrated that 1,25(OH)2D3 and its analogues significantly reduce PTH in non-dialysis patients with mildly elevated PTH in CKD3a-5 (Wang et al., 2014; Li et al., 2015). However, there was no significant improvement in cardiac function, but obvious hypercalcemia appeared in these patients, and mild to moderate elevation of PTH was considered to be an adaptive response. Therefore, 2017 KDIGO Chronic Kidney Disease–Mineral and Bone Disorder (CKD-MBD) Guideline emphasizes dynamic monitoring of PTH. 1,25(OH)2D3 and its analogues are considered to be appropriate only for the treatment of severe and progressive hyperparathyroidism and for patients with progressively elevated parathyroid hormone levels or consistently above the normal upper limit should be assessed for hyperphosphoemia, a high-phosphorus diet, hypocalcemia, and vitamin D deficiency for individualized treatment (KDIGO, 2017). The recommendation referred to in the guidelines not to routinely use calcitriol or its analogues in CKD3a-5 was not met with consensus among working Group members, however, the J-David trial did not support the application of VDRAs to prevent CVD in patients with ESKD and relatively low PTH as well (Shoji et al., 2018).

Cinacalcet, a positive allosteric regulator of CaSR, significantly enhances the sensitivity of CaSR-mediated intracellular signaling pathway to Ca2+i and can inhibit the release of parathyroid hormone (Nemeth & Bennett, 1998). It is clinically effective for both secondary hyperparathyroidism (Messa et al., 2008) and parathyroidism (Nemeth & Shoback, 2013). Leach K et al. showed that calcimimetics have ligand-biased modulation, and cinacalcet is biased towards Ca2+i mobilization and inositol 1-phosphate (IP1, a stable metabolite of IP3) accumulation. It can be considered that cinacalcet binds to CaSR and favours a receptor conformation that preferentially couples to intracellular Gq signaling pathway, promoting Ca2+i mobilization and inhibiting parathyroid hormone release through the PLC-IP pathway. Cinacalcet can also inhibit or even reverse parathyroid hyperplasia (Miller et al., 2012). In this trial, p21 expression was increased in parathyroid tissues of uremic rats treated with cinacalcet compared with control rats. Therefore, a potential mechanism by which cinacalcet prevents proliferation of parathyroid cells in uremic rats is by increasing p21. However, it is possible that changes in p21 are not the cause but may be a response to changes in cell proliferation. After calcimimetics treatment, the expression of 1α-hydroxylase gene in parathyroid gland was further increased by 42%, and the expression of 24-hydroxylase gene was decreased by 2.2-fold, leading to a decrease of about 53% in parathyroid hormone gene expression (Ritter et al., 2012). The expression of CaSR and VDR genes in uremic rat PTG within 2 weeks was increased rapidly by calcimimetics (Riccardi & Martin, 2008), and promoted the inhibitory effect of 1,25(OH)2D3 or VDRA analogues on PTH synthesis and parathyroid cell proliferation, resulting in the regression of PTG proliferation. The clinical use of cinacalcet in hyperparathyroidism has been limited by its propensity to induce hypocalcemia, in part due to activation of CaSR in the thyroid gland and stimulation of calcitonin release. CaSR allosteric modulators that selectively bias signaling toward pathways that mediate desired effects (e.g., parathyroid hormone (PTH) inhibition) rather than those that mediate adverse effects (e.g., elevated serum calcitonin) may provide better treatment.

In the clinical situation, the majority of patients with SHPT will, despite previous long-term uremia, present a significant fall in plasma PTH after kidney transplantation. This may be due to an improvement in glomerular filtration rate (GFR). The normalization of parathyroid function is related to the inhibition of parathyroid hyperplasia and the reduction of parathyroid hormone synthesis and secretion. Interestingly, parathyroid hormone secretion decreased significantly after reversal of uremia by homogeneic renal transplantation, while the expression of CaSR and VDR genes in parathyroid glands remained decreased. This might indicate the existence of a secretory mechanism in the parathyroid cells that is not coupled to CaR and which responds to reversal of uremia or to the simultaneous normalization of plasma calcium and phosphorus levels. The model of parathyroid hyperplasia clearly shows that parathyroid hyperplasia can be controlled in non-uremic animals. The study of Taniguchi et al. suggested that in patients with persistent secondary hyperparathyroidism after renal transplantation, the fate of hyperplastic glands was related to their proliferation pattern, and the diffuse hyperplastic glands had a strong tendency to fade. The remaining large nodular hyperplasia indicates little reversibility (Taniguchi et al., 2006). Thus, the restoration of active vitamin D production in the transplanted kidney is thought to play a major role in its process, and more importantly, the reactivity of diffuse hyperplasia to active vitamin D is intact, whereas it is absent in nodular hyperplastic glands. The work of Brown et al. previously showed that calcium regulates the VDR messenger RNA expression in rat parathyroid glands (Brown et al., 1995). However, in the study by M et al., plasma calcium levels normalized after kidney transplantation in rats, while VDR mRNA levels in parathyroid glands continued to decrease (Lewin et al., 2002). This difference may be due to the fact that normalization of plasma calcium after experimental renal transplantation is very rapid (Lewin et al., 1997) rather than a response to chronic dietary therapy or a pharmacological effect of 1,25 (OH)2D3. Another possible explanation is that 1,25 (OH)2D3 activity in the parathyroid glands of kidney transplanted rats is improved by cessation of hypocalcaemia, despite persistently low VDR expression (Sela-Brown et al., 1998; Garfia et al., 2002). However, much remains to be understood about the mechanisms that are controlling the function of the hyperplastic glands after reversal of uremia by a successful kidney transplantation.

In patients with chronic renal failure, calcium, phosphorus, parathyroid hormone and 1,25(OH)2D3 have biological interactions that promote and inhibit each other. Therefore, it may be wrong to interpret the effect of a single drug without taking into account differences in the associated combined interventions of CKD-associated mineral metabolism. Further observation and randomized studies are required to determine who should receive VDRA treatment, the biochemical target level of treatment, and to avoid intervention too early, too late, utilising the incorrect adjuvant therapy, or administering it to the wrong patient.
